# Supplementary material for: Assessing the impact of CHARM2, a family planning program on gender attitudes, intimate partner violence, reproductive coercion, and marital quality in India
Source: PLOS Glob Public Health. 2024 May 21;4(5):e0003220. doi: 10.1371/journal.pgph.0003220 (PMC11108161; doi:10.1371/journal.pgph.0003220)
Supplement: Attachment — Submitted filename: Response to reviewers 2.22.24.docx [file pgph.0003220.s005.docx]

| Reviewer comment | Response | Page, line nos. |
| --- | --- | --- |
| Data were analysed statistically. Data analyses were relevant and consistent to support the claims. However, it would be good to check the tools by a statistician to confirm their sufficiency and whether other evidence is required. | We have clearly outlined our methods, specified the tools, and described the analytic procedures. Our broader study team includes a senior biostatistician, who was informed of and advised our approach to evaluating intervention effects; the approach utilized was deemed appropriate and sufficient. We are happy to respond to any further specific queries regarding our tools. |  |
| The paper is considered suitable for publication but reporting the study findings following the CONSORT checklist or justifying how the 25 items of the CONSORT checklist are considered is highly recommended. | We have included a CONSORT flow diagram. We have not outlined the results using the CONSORT checklist as this is a secondary analysis of trial results. A primary trial paper has already been published and is linked in the main body of the paper. |  |
| Acronyms should be expanded in words/ phrases when they are used for the first time. | We apologise for this oversight and have ensured we expand acronyms for the first time throughout the text. | Pg. 3, 64. |
| In the methods, you refer a couple of times to other articles (e.g. “Additional details  are available in the study protocol (20, 21)” and “More details on the intervention are available elsewhere (20, 21)”). I advise you to elaborate more on your methods in this manuscript. You cannot expect readers to return to your earlier published articles to review the methods. This paper should be complete and clear. | Thank you for your feedback. We have retained a single reference for readers who would like to review the main trial results and have ensured that the methods section provides sufficient detail. | Pg. 5, 119. |
| P5 line 115-116: I understand that the clusters were randomized, so the couples were included depending on the cluster where they were living. This might give bias which you should describe in the limitations.- and response bias which should be described in the limitations. | While the characteristics of couples likely differ between geographic clusters, randomization at the cluster level minimized potential bias with regard to these differences in receipt of treatment versus control conditions. Furthermore, we evaluated whether the populations randomized to control versus intervention differed in terms of several sociodemographic characteristics and controlled for those factors as well as any factors associated with loss to follow-up in all adjusted analyses to mitigate further potential bias introduced by such differences. Please refer to the methods section, lines 247-257, for additional detail on this control.  We have addressed response bias in the limitations section: “*All measures relied on self-report and are therefore subject to disclosure and underreporting bias. We attempted to mitigate social desirability bias by using researchers who were external to the program, trained in gender sensitivity and emphasised the confidentiality of all answers*.” | Pg. 9, 247-57.  Pg. 18, 423-26. |
| P5 line 126-127: how were the participating couples recruited from the included clusters? Were couples selected by the HCP and face-to-face asked to participate? Was every couple from sept 10 screened for inclusion by an independent recruiter, or could the treating HCP preselect risking selection bias? Please elaborate on this. | We have added this information to the manuscript for clarification: “*Participants were recruited in a two-stage process. First, study staff screened all households in the cluster for couples meeting study eligibility criteria by approaching households face-to-face and compiling a list of all eligible couples. Second, we used a random number generator to determine which couples from the eligible list to approach for enrolment. This enrolment was conducted at the participant’s home via face-to-face recruitment.”* | Pg 6, 146-51 |
| P6 Line 148-150: not clear.  *Was the final session with the couple, or was the final session individual? Or did this depend on the preference of the participating couple? Then this should be described in the results.  *Please describe the aim of this final session with the couple.  *If this final session was done with the couple, response bias will be a possible limitation since people might not dare to speak freely and give desirable answers, so this should be described in the limitations. | We have added text to clarify that the final session was delivered to both husband and wife: “*A male or female provider provided a final session for couples (both husband and wife attended this session together), who delivered the individual sessions for either spouse based on availability or participants’ preference.*”  We have added the following information to describe the aim of the final session with couples: “*In addition to the individual sessions, a joint session consolidated the main takeaways of the intervention around methods of family planning and the importance of communication and joint decision-making*.”  We would like to clarify that the final session refers to the last session of the intervention. Data were not collected from either the husband or the wife during the intervention, so there is no issue of response bias. | Pg 7, 1164-65.  Pg 7, 184-86. |
| P7 line 165-166: what was the reason not to include RC in the counselling of men? | We apologise for the error. The topic of RC was discussed with male participants. We have added this sentence to clarify: “*Both women and men received input on domestic violence, its causes, reproductive coercion, and steps that can be taken to prevent violence*.” | Pg 7, 182-186. |
| Page numbering (see table 2): the page numbering restarts at 1. Please adjust this to continuous numbering. | We have rectified this issue in the current version. |  |
| Line 307: you write 12 months F/U, but this should be 18 months F/U. Please adjust. | We asked participants to recall their experiences of RC over the past 12 months. It is specifying the recall period rather than the point at which data was collected. |  |
| Please add an extra heading “Recommendations for future studies” after the discussion and conclusion (which is line 396-403). You could for example describe a study on behavioral change since this did not change in your study, as pointed at in line 353. | We have added a heading for conclusion rather than recommendations as this is the last paragraph of the paper and we have summarised the main results of the study in this section. | Pg 18, 427. |
| Please make sure that your datasets are part of the manuscript. | We have uploaded our analysis dataset in this round of submissions. |  |
| It appears that there are about four outcome variables here and I would think the data used here could have provided two manuscripts. This would have allowed the authors to thoroughly engage the results more. | We appreciate your feedback. We wanted to present secondary analyses on gender equity related factors in one manuscript. We have attempted to present results in a coherent manner. |  |
| It will be important to explain the terms as used in the paper. For example, what is reproductive coercion, marital quality and gender transformative and gender equity. This allows the reader to understand from the onset what the terms seek to explain or measure. | Thank you for the suggestion. We have added a definition for reproductive coercion: “*Reproductive coercion refers to behaviours that interfere with a woman’s autonomous decision-making around reproductive health. It includes birth control sabotage, pregnancy coercion, or controlling the outcome of a pregnancy (5)*.” We also added a line to explain marital quality: “*Marital quality is a predictor of a couple's well-being and is often used interchangeably with terms such as satisfaction, adjustment, well-being, happiness, and success (27).”*  We had described gender-transformative, and gender-equity-focused interventions on Page 3, lines 75-78: “*GE-focused and gender-transformative interventions typically examine and disrupt harmful gender norms to reduce gender inequalities and improve health outcomes (8). These interventions encourage critical reflection on gender norms, promote shared decision-making among couples, and increase women’s access to resources*.” | Pg 3, 66-68.  Pg 9, 221-23.  Pg 3, 78-81. |
| Can you provide a little more information about the research area? Apart from the fact that Maharashtra was chosen due to lower than state level modern contraceptive use and higher male-female sex ratio? For instance, what are traditional gender norms/attitudes/socialization/ women’s empowerment/ women’s level of education in the area and how will these influence the uptake of the intervention. In doing this you are able to add more meaning to the results during the discussion. | We have added the following sentences to provide additional context: “*In rural Pune, female illiteracy is 27%, and the child sex ratio is 833 girls per 1000 boys (indicative of son preference)*”. | Pg 5, 118-19. |
| Line 80 to 81 “…gender equitable attitudes in studies conducted among a sample of participants in India (12)…..” rather than “….gender equitable attitudes in India (12)…..” | We made this change: “*GE-focused FP programs positively impacted attitudes in studies conducted among a sample of participants in India…*”. | Pg 4, 83-84. |
| Line 90-91 “…significantly improved contraceptive communication, modern contraceptive use among couples, REDUCED women’s experiences of sexual IPV and IMPROVED men’s equitable towards gender norms.” | We made this change: “…significantly improved contraceptive communication, modern contraceptive use among couples, reduced women’s experiences of sexual IPV, and improved men’s attitudes toward gender norms…” | Pg 4, 93-97. |
| It would be important to throw some light on why CHARM reduced women’s experience of IPV but in CHARM2 the intervention did not appear to impact sexual IPV experience. Were there more educational sessions in CHARM than CHARM2. If there were why were the number of educational sessions reduced in CHARM2? Was it just about sample selection? Or something else? | Both studies were conducted in rural Maharashtra and had similar content. The CHARM study was conducted in a tribal area with higher rates of IPV and alcohol use and greater levels of poverty. The CHARM2 study was conducted in a more agriculturally rich area, and the sample included more middle-class landowners. The CHARM2 sample showed lower levels of IPV than that seen in Maharashtra as a whole. Further, the period of time between CHARM and CHARM2 saw an increase in mass media communications programs designed to shift norms related to IPV, as well as increased access to media in rural areas (Wang & Singhal, 2018). For example, in 2019-21, 54% of women reported access to their own mobile phone as compared to 45% in 2015-16 (IIPS & ICF, 2021). These factors may have led to a difference in results between studies. We have not noted this in the paper as we wanted to focus on the results of CHARM2 rather than compare the results of CHARM and CHARM2. In the discussion section, we have outlined several factors, including the low prevalence of IPV leading to a ceiling effect, insufficient dosage and content on IPV, and the stressors associated with the COVID-19 pandemic that may explain why the CHARM2 intervention did not have an impact on IPV.  Hua Wang & Arvind Singhal (2018) Audience-centered discourses in communication and social change: the ‘Voicebook’ of Main Kuch Bhi Kar Sakti Hoon, an entertainment-education initiative in India, *Journal of Multicultural Discourses*, 13:2, 176-191, DOI: 10.1080/17447143.2018.1481857.  International Institute of Population Sciences (IIPS) and ICF (2021). National Family Health Survey (NFHS-5), 2019-21: India: Volume I. Mumbai, IIPS. | Pg 16, 375-416. |
| Line 93 “……reversible contraceptives methods and women participation” Is it women’s participation in the intervention? | We have modified the sentence to note the intention to increase women’s participation in the intervention. | Pg 4, 94-97. |
| Line 97….please clarify why a measure of marital quality was included to assess any potential “BACKLASH” | We agree that this sentence was confusing, and we have removed it. |  |
| Line 138 “If both PARTNERS were present, consent was obtained from THEM…..” rather than “If both members of a couple were present, consent was obtained from both partners..” | We have made the required change: “*If both members of a couple were present, consent was obtained from them simultaneously by gender-matched study staff*.” | Pg 6, 152-53. |
| Line 220 to 221……. What about others who do not belong to the caste stated here? Could you write something about them? For example, are they economically more empowered? How are they referred to? | Participants who belonged to socioeconomically minoritised lower caste groups are coded as 1, and all other participants who belong to higher caste groups are coded as 0 and fall in the reference category.  We noted this in the method section: “*Participants were asked to denote their caste, and the caste status variable was recoded as a binary variable to identify participants who belonged to Scheduled Castes, Scheduled Tribes, or Other Backward Classes (0=no, 1=yes). SC/ST/OBC individuals are a socioeconomically minoritized group in India*.” | Pg 10, 239-42. |
| Line 248 “….between intervention and CONTROL GROUPS at baseline.” rather than “…between intervention groups”? | We have made the requested change: “*There were some significant differences between intervention and control groups at baseline*.” | Pg 11, 269. |
| 307 “…18-months follow-ups” rather than “… rather than 12-month follow-up.” | This sentence has been modified: “However, this difference was not sustained at the 9 or 18-month follow-ups.” | Pg 14, 332. |
| The discussion looks well done but it appears that the authors dwell a lot more on the outcomes that may not have had the most impact from the intervention. Could the authors in the first paragraph of the discussion discus further possibly, why and how the intervention had that kind of impact on attitudes towards wife-beating, men’s equitable gender attitude and marital satisfaction? Why did the intervention have such effects. | Thank you for this excellent suggestion. We have added the following sentences to the first paragraph of the discussion: “*CHARM2 encouraged participants to reflect on gender inequalities and traditional social norms underlying IPV, RC, and son preference and emphasized the importance of shared decision-making and healthy communication for a happy married life. These factors may have led to a shift in attitudes towards more egalitarian gender norms*.” | Pg 16, 368-71. |
